# Transient Receptor Potential Channels and Inflammatory Bowel Disease

**DOI:** 10.3389/fimmu.2020.00180

**Published:** 2020-02-20

**Authors:** Yiding Chen, Jingxi Mu, Min Zhu, Arjudeb Mukherjee, Hu Zhang

**Affiliations:** ^1^Department of Gastroenterology, West China Hospital, Sichuan University, Chengdu, China; ^2^Centre for Inflammatory Bowel Disease, West China Hospital, Sichuan University, Chengdu, China; ^3^West China School of Medicine, Sichuan University, Chengdu, China

**Keywords:** transient receptor potential channels, gastrointestinal tract, neurogenic inflammation, immune cells, inflammatory bowel disease

## Abstract

The transient receptor potential (TRP) cation channels are present in abundance across the gastrointestinal (GI) tract, serving as detectors for a variety of stimuli and secondary transducers for G-protein coupled receptors. The activation of TRP channels triggers neurogenic inflammation with related neuropeptides and initiates immune reactions by extra-neuronally regulating immune cells, contributing to the GI homeostasis. However, under pathological conditions, such as inflammatory bowel disease (IBD), TRP channels are involved in intestinal inflammation. An increasing number of human and animal studies have indicated that TRP channels are correlated to the visceral hypersensitivity (VHS) and immune pathogenesis in IBD, leading to an exacerbation or amelioration of the VHS or intestinal inflammation. Thus, TRP channels are a promising target for novel therapeutic methods for IBD. In this review, we comprehensively summarize the functions of TRP channels, especially their potential roles in immunity and IBD. Additionally, we discuss the contradictory findings of prior studies and offer new insights with regard to future research.

## Introduction

Inflammatory bowel disease (IBD) is a chronic relapsing GI inflammatory disorder, comprising of Crohn's disease (CD) and ulcerative colitis (UC). It is acknowledged that IBD is related to inappropriate immunity, commensal bacteria, genetics, and environmental factors. The exact pathogenesis of IBD, however, remains unknown (1). Nowadays, various receptors in the gastrointestinal (GI) tract are proposed to play a role in the pathophysiology of IBD, amongst which transient receptor potential (TRP) ion channels have been identified and are considered to be potentially effective. TRP channels are polymodal ion channels that serve as sensors for chemical noxious and physical stimuli. These channels are widely distributed in the GI tract and exert various effects, contributing to the somatic and visceral nociception and the maintenance of physiological function of the GI tact (2). The activation of TRP channels can evoke neurogenic inflammation, namely the inflammation initiated by the local release of immunomodulatory neuropeptides, including calcitonin-gene-related peptide (CGRP) and substance P (SP) released by unmyelinated afferent neurons (3, 4). Some TRP channels are also expressed in multiple immune cells, and are primarily responsible for modulating actions, such as cytokine release, cell migration, and phagocytic activity (2). Therefore, numerous studies have indicated that TRP channels are mainly involved in the visceral hypersensitivity (VHS) and immune pathogenesis of IBD due to their comprehensive functions of sensors and immunomodulators. Different subtypes of TRP channels seem to have distinct effects. Here, we briefly review the correlation between TRP channels and IBD with a focus on TRPV1, TRPA1, TRPV4, TRPM2, and TRPM8, which have been documented to be the most relevant TRP channels in IBD.

## TRP Channels in the GI Tract and Related Neuropeptides

In the GI tract, TRP channels are mainly expressed on the extrinsic primary afferent nerves with some on epithelial, endocrine cells, and intrinsic enteric neurons (5, 6). Intriguingly, 97% of TRPA1-positive (TRPA1^+^) afferents co-express TRPV1, and 30% of the TRPV1-positive (TRPV1^+^) neurons co-express TRPA1, hinting at the potential interaction between the two channels (5, 7). Capsaicin is a significant agonist for TRPV1 with an exquisite selectivity and allyl isothiocyanate (AITC), the pungent ingredient in garlic, is the prototypical agonist for TRPA1 (8, 9). TRPV4 colocalizes with TRPV1, TRPA1, and protease-activated receptors 2 (PAR-2) in the GI tract, in response to strong acidosis, hypotonicity, warmth, and mechanical stimuli (10, 11). TRPM2 is sensitive to heat stimulus while TRPM8 is essential to cold-induced pain (12). Most of TRP channels are non-selective cation channels and show the permeability to calcium ion (Ca^2+^). Upon stimulation, TRP channels in afferents can lead to autonomic reflex responses by transmitting signals to the central nervous system. Meanwhile, TRP channels can transduce sensory signal of G-protein coupled receptors (GPCRs) based on the phosphorylation sites in N-terminus for serine and threonine protein kinases, such as protein kinase A (PKA) and protein kinase C (PKC) (11, 13).

Additionally, TRP channels in the GI tract can mediate the crosstalk between the nervous and immune systems by modulating the release of neuropeptides. TRPV1, TRPA1, and TRPV4 are especially often found to colocalize with CGRP and SP (6, 14).

CGRP, which is generated from the alternative RNA processing of the gene for calcitonin, serves as a potent peptide vasodilator and is involved in the transmission of nociception (15). CGRP plays a protective role in the inflammation and inhibits the capacity of immune cells. For dendritic cells (DCs) and macrophages, CGRP could restrain their ability in the presentation of antigens and the secretion of pro-inflammatory cytokines, such as tumor necrosis factor alpha (TNF-α) (16, 17). CGRP also downregulated DCs' responses to Toll-like receptor 4 (TLR4), a receptor for lipopolysaccharides (LPS) which is an abundant outer wall glycolipid of Gram-negative bacteria (18). CGRP can exert an inhibitory effect on the activation and chemotaxis of neutrophils (19), and inhibit neutrophil-mediated killing of bacteria mainly through suppressing the activity of the bactericidal enzyme myeloperoxidase (MPO) in a dose-dependent manner (20). CGRP was found to restrain group 2 innate lymphoid cells proliferation and type 2 innate immune responses (21), and be required for the induction of protective innate type 17 immunity after the activation of cutaneous TRPV1^+^ neurons (22). Furthermore, CGRP could induce the upregulation of interleukin (IL)- 10 and was beneficial in preserving mucosal integrity and limiting tissue damage (16). These observations demonstrate the negative regulatory role of CGRP in innate immunity and the benefits of CGRP in the GI tract. Conversely, GRCP was reported to be capable of stimulating T-cell migration and promoting the release of interferon gamma (IFN-γ) and IL-2 from T-helper cells (23, 24). In IBD patients, the expression of CGRP in the colonic mucosa was significantly increased and was closely associated with the severity of disease (25, 26). Therefore, CGRP might also play a part in the pro-inflammatory process.

SP belongs to tachykinin family. The receptor for SP is the neurokinin 1 receptor (NK-1R) (15). Similar to GCRP, SP serves as a potent vasodilator and the SP-induced vasodilatation is based on nitric oxide (NO) release (27). Of interest, high-dose CGRP was reported to restrain the SP-evoked vasodilation but facilitate SP-evoked plasma protein extravasation (28), suggesting a crosstalk between CGRP and SP. The expression of SP and NK-1R has been well-documented in DCs, monocytes, eosinophils, neutrophils, mast cells, natural killer cells, and T cells, enabling SP to regulate functions of different types of immune cell (29). SP also modulated the immune response to microbial infection (29). It was recently demonstrated that SP could promote the migration and activation of mast cells, inducing the release of multiple pro-inflammatory cytokines and chemokines (30). Noteworthy, SP directly caused the secretion of IL-8 in human colonic epithelial cell lines (31), hinting at the potential pro-inflammatory role of SP in intestine. SP was detected to be elevated in tissue extracts from the colon and rectum of IBD patients, and the level of SP was correlated with disease activity (32). However, in animal studies, SP ameliorated dextran sulfate sodium (DSS)-induced colitis by promoting the enrichment of M2 macrophages and regulatory T cells, or maintaining barrier structure and regulating immune response (33, 34). Such results remind us of the possible protective effect of SP in colitis. Taking these contradictory observations with regard to the properties of CGRP and SP into consideration, we can conclude that the neurogenic inflammation triggered by TRP channels has bidirectional functions on immunity and colitis. However, the exact function of neuropeptides on a certain physical or pathological condition has not yet been discovered and further studies are required.

## TRP Channels in Immune Cells

Besides the roles in nervous system of the GI tract, TRP channels are also expressed in immune cells and directly contribute to immune responses. In bone marrow-derived macrophages, the TRPV1 expression was increased and intracellular Ca^2+^-transients were triggered after oxidized low-density lipoprotein (ox-LDL)-stimulation (35). TRPV1 could dose-dependently modulate the level of inducible NO synthase in stimulated peritoneal macrophages through the inhibition of nuclear factor kappa B (NF-κB), thus influencing the secretion of pro-inflammatory cytokines involved in this pathway (2). In a sepsis model, the LPS-stimulated peritoneal macrophages showed an impaired phagocytosis when TRPV1 was knock-out (36), suggesting the putative role of TRPV1 to potentiate macrophages. In CD4^+^ T cells, TRPV1 was associated with T cell receptor (TCR) and facilitated TCR-induced Ca^2+^ inflow (37), and the activity of CD4^+^ T cells was impaired via the inhibition of TRPV1 (38). The activation of TRPV1 was also reported to enhance leukocyte rolling and adhesion (39). These data indicated the possible pro-inflammatory properties of TRPV1 in immune cells. Intriguingly, TRPV1 activation can trigger the production of the endocannabinoid anandamide, which increases the level of regulatory CX3CR1 (hi) macrophages in the gut and enhances their immunosuppressive activity (40). In DCs, TRPV1 mediates the downregulation of TLR4/NF-κB signaling pathway that leads to the maturation of DCs (41). A recent research concerning lethal Staphylococcus aureus pneumonia stated that the activation of TRPV1^+^ nociceptors by capsaicin could suppress cytokine release, inhibit the recruitment and surveillance of neutrophils, and alter lung γδ T cell numbers; thus impairing lung bacterial clearance (42). As discussed above, TRPV1 is an important immunomodulator that regulates the activation and function of immune cells.

As for TRPA1, its expression was increased in stimulated T cells, and TRPA1 was vital for the T cell activation and release of cytokines like TNF-α, IFN-γ and IL-2 (43). TRPA1 also expresses in mast cells and DCs (44). In TRPA1-knockout (*Trpa1*^−/−^) mice, mast cells, leukocytes, and T cells, together with the expression of IL-1β, IL-6, IL-17, IL-22, and IL-23 were decreased in the lesions of skin (45), indicating the ability of TRPA1 to induce inflammation through these immune cells. In addition to its pro-inflammatory function, the activation of TRPA1 could suppress the pro-inflammatory effect of LPS-stimulated peritoneal macrophages by decreasing the level of NO, which is an abundant pro-inflammatory mediator (46). Taken together, TRPA1 has the ability to regulate immune cells in diverse manner. The crosstalk between TRP channels and bacteria is noteworthy. It was discovered that LPS interacted with TLR4 on the TRPV1^+^ afferent neurons. This then activated or sensitized TRPV1 via its phosphorylation binding sites through PKC, thus resulting in an increased release of CGRP (17, 47). Antagonists for TRPV1 and CGRP could reverse LPS-induced motility disturbance of the intestine (48). Another study showed that a probiotic bacterium named Lactobacillus reuteri and its condition medium dose-dependently reduced the capsaicin- and distension- evoked firing of jejunal spinal afferents in mice (49), revealing the engagement of afferents in bacteria-induced GI sensory-motor dysfunction. TRPA1 in nociceptive neurons could be sensitized by LPS in a TLR4-independent manner during inflammation, causing pain, CGRP release, and vasodilation (50). Therefore, it was hypothesized that TRP channels may be able to directly or indirectly interact with microbiota or their products in the gut, thus influencing the release of neuropeptides and contributing to the maintenance of gut homeostasis.

The activation of TRPV4 increased intracellular Ca^2+^ concentration in LPS-treated macrophages and potentiated macrophage (51), while downregulation of TRPV4 subsequently impaired the phagocytosis of macrophages (52). In neutrophils, TRPV4 was essential for inflammatory responses, such as the neutrophil adhesion, chemotaxis, and formation of reactive oxygen species (53). TRPV4-mediated Ca^2+^ influx in T cells was also capable of inducing the proliferation of T cells and the secretion of TNF-α, IFN-γ, and IL-2 *in vitro* (54). Recent data revealed that TRPV4 could promote the phagocytosis of mouse CD11c^+^ bone marrow-derived cells (55). These findings clearly highlight the critical pro-inflammatory role of TRPV4 in immune cells.

Regarding TRPM2, it was demonstrated that the lack of this channel in LPS-stimulated monocytes cell line reduced the release of TNF-α, IL-6, IL-8, and IL-10 (56). TRPM2-associated Ca^2+^ signaling was essential in the transmigration and cytotoxicity of neutrophils (57, 58), the proliferation of T cells, and the release of pro-inflammatory cytokines (59). For TRPM8, the activation by menthol in murine peritoneal macrophages increased IL-10 expression and decreased TNF-α release, thus exerting an anti-inflammatory effect (60). TRPM8-knockout (*Trpm8*^−/−^) peritoneal macrophages exhibited an impaired phagocytic activity while the phagocytosis was enhanced in WT peritoneal macrophages after the activation of TRPM8 (60). Consistently, the activation of TRPM8 was reported to restrain the release of pro-inflammatory mediators in monocytes and lymphocytes, and *Trpm8*^−/−^ CD11c^+^ DCs showed hyperinflammatory responses to TLR-stimulation (61, 62). In T cells, the inhibition of TRPM8 suppressed murine T-cell activation and the release of IL-2 and IL-6 (63). Overall, TRPM2 appears to potentiate inflammatory effects of immune cells while TRPM8 often performs anti-inflammatory roles.

## TRP Channels in Inflammatory Visceral Hypersensitivity of IBD

Due to their immunomodulatory function via neuropeptides and immune cells, TRP channels are associated with GI immunity and inflammation. Notably, it is found that their expression has been altered in IBD patients and colitis models (Table 1), suggesting an involvement of TRP channels in IBD. In particular, IBD patients are associated with a visceral hypersensitivity (VHS), which is featured of an aberrant and chronic visceral pain (5, 12). As visceral nociceptors, TRP channels are proposed to be responsible for VHS in IBD. Since TRP channels serve as secondary transducers for GPCRs, some mediators that act on GPCRs subsequently activate or sensitize TRP channels, resulting in aberrant sensation. Through this mechanism, pro-inflammatory mediators secreted during colitis, such as bradykinin, serotonin (5-hydroxytryptamine, 5-HT), cytokines, adenosine triphosphate, prostaglandins, and epinephrine can lead to the inflammatory VHS (5). A number of researches have been conducted to explore the definite role of TRP channels in colitis and VHS (Table 2).

**Table 1:** The expression of TRP channels in colonic tissue of IBD patients and colitis models.

	**TRPV1**	**TRPA1**	**TRPV4**	**TRPM2**	**TRPM8**
**IBD patients**					
UC	UP (64–68) NS (69) DOWN (70, 71)	UP (68)	UP (70, 72) NS (73)	NA	NA
CD	UP (64–66) DOWN (68, 71)	UP (68, 71, 74, 75)	UP (72, 76) NS (73)	NA	UP (77)
**Animal models**					
DSS-treated mice	UP (68, 78–80) NS (71)	UP (71)	UP (73, 81)	NA	UP (77, 82)
DSS-treated rats	UP (83)	NA	NA	NA	NA
TNBS-treated mice	UP (84)	NA	UP (72)	NA	UP (77, 82)
DNBS-treated mice	NA	UP (85)	NA	NA	NA
TNBS-treated rats	UP (86) NS (87)	UP (88, 89)	NA	UP (90)	NA
Mustard oil-treated mice	NS (91)	UP (91)	NA	NA	NA

*UC, ulcerative colitis; CD, Crohn's disease; DSS, dextran sulfate sodium; TNBS, 2,4,6-trinitrobenzenesulfonic acid; DNBS, dinitrobenzene sulfonic acid; UP, upregulated; DOWN, downregulated; NS, no significant difference; NA, not available*.

**Table 2:** The function of TRP channels in VHS of colitis models.

**Pro-VHS function**			
**TRP channel**	**Colitis model**	**Result**	**References**
TRPV1	DSS-treated mice	Increased VHS which could be enhanced by the agonist for TRPV1	(92)
	DSS- and TNBS- treated rat	Increased VHS which could be relieved by the antagonist for TRPV1	(83, 86, 87, 93)
	Trpv1^−/−^ mice with DSS-induced colitis	Decreased VHS compared to WT mice	(78)
TRPA1	DSS-treated mice	Increased VHS which could be enhanced by the agonist or be relieved by the antagonist for TRPA1	(94)
	TNBS-treated rat	Increased VHS which could be enhanced by the agonist or be relieved by the antagonist for TRPA1	(88, 89, 95, 96)
	Trpa1^−/−^ mice with TNBS-induced colitis	Decreased VHS compared to WT mice	(97, 98)
TRPV4	TNBS-treated mice	Increased VHS which could be relieved by the antagonist for TRPV4	(72)
	Trpv4^−/−^ mice	Decreased VHS compared to WT mice	(99)
TRPM2	TNBS-treated rat	Increased VHS which could be relieved by the antagonist for TRPM2	(90)
	Trpm2^−/−^ mice with TNBS-induced colitis	Decreased VHS compared to WT mice	(90)
TRPM8	DSS- and TNBS- treated mice	Increased VHS which could be enhanced by the agonist or be relieved by the antagonist for TRPM8	(82)
	Trpm8^−/−^ mice	VHS was only decreased under higher level of stimuli compared to Trpv1^−/−^ and Trpv4^−/−^ mice	(100)
**Anti-VHS function**			
TRPM8	WT mice	The function of TRPV1 and TRPA1 was inhibited by TRPM8 activation	(101)
	TNBS-treated rat	Increased VHS which could be relieved by the agonist for TRPM8	(102)

*VHS, visceral hypersensitivity; DSS, dextran sulfate sodium; TNBS, 2,4,6-trinitrobenzenesulfonic acid; WT, wild-type*.

### TRPV1

TRPV1 channel is closely linked to VHS. It was found that some patients with quiescent IBD still complained about abdominal pain, and the severity of their symptoms was correlated to the increased TRPV1^+^ fibers in colonic mucosa (64). In animal studies, the behavioral responses to intracolonic capsaicin administration and the expression of spinal cord neuronal c-Fos, which is a marker of neuronal excitation, were increased in DSS-treated mice (92). Yang et al. (83) reported that an oral administration of curcumin, which is clinical valuable for the treatment of IBD (103), in DSS-treated rats could significantly ameliorate visceral hyperalgesia through inhibiting phosphorylation of TRPV1, indicating a nociceptive effect of TRPV1. Likewise, Phillis et al. (93) revealed that TRPV1 antagonist remarkably reduced the mechanosensory response to the stimulus in a dose-dependent manner in rats with DSS-induced colitis. Visceral hyperalgesia and increased visceromotor response (VMR) were also confirmed in rats with 2,4,6-trinitrobenzenesulfonic acid (TNBS)-induced colitis (86, 87), while TRPV1 antagonist (JYL1421) could prevent and relieve the VHS (86). Additional studies exhibited that TRPV1-knockout (*Trpv1*^−/−^) mice conferred a resistance to colorectal distension (78, 104), and VHS was enhanced by inflammatory mediators, such as bradykinin, 5-HT, histamine, and prostaglandin E2 (PGE2) in WT mice but not in *Trpv1*^−/−^ mice (12). SP was demonstrated to enhance the sensitivity and function of TRPV1 in DSS-induced colitis and *in vitro* (78), suggesting the engagement of neuropeptides in VHS. Noteworthy, the augmented activity of pelvic nerve afferents after TRPV1 activation in DSS-treated rats was more prominent on the first day post DSS-treatment, in comparison to the eighth day (92). Similarly, the levels of TRPV1 and TRPA1 messenger RNA (mRNA) in mice were upregulated in mustard oil (MO)-induced colitis within 6 h but decreased 24- and 72-h after MO-injection (91). Therefore, it can be hypothesized that the excitatory mechanism modulated by TRPV1 mainly particulate during early stage of experimental colitis.

### TRPA1

TRPA1 could contribute to colorectal contraction and enhanced VMR to intracolonic AITC, which were detectable in TNBS-induced colitis. These actions could be suppressed by intrathecal pretreatment with a TRPA1 antisense oligodeoxynucleotide, and were absent in *Trpa1*^−/−^ mice (88, 89, 97, 98). Likewise, AITC enhanced the sensitivity of colon and the expression of c-Fos in spinal cord of DSS-treated mice (94). During TNBS-induced colitis, the production of hydrogen peroxide (H_2_O_2_) was enhanced due to the infiltration of white blood cells and the presence of oxidative stress. The increased H_2_O_2_ then activated TRPA1 and led to the hypersensitivity of VMR (95). The aberrant GI motility might result from the effects of PGE2 induced by TRPA1 activation (105). Similarly, inflammatory mediators, such as bradykinin and 5-HT could lead to an increased visceral mechano-sensitivity in a TRPA1-associated manner (97, 106). These data suggest a close link between TRPA1 and pro-inflammatory cytokines, both of which contribute to visceral hyperalgesia. Of interest, Vermeulen et al. (96) reported that a combined application of antagonists for TRPV1 and TRPA1 could reduce the VMR more effectively in TNBS-induced colitis, in comparison to targeting either TRPV1 or TRPA1 alone. Such evidence appears to provide an inspiring therapeutic method for inflammatory VHS.

### TRPV4

TRPV4 is vital for a mechanically-evoked visceral pain in the GI tract (76). It was found that TRPV4 co-expressed with PAR-2 and pretreatment of PAR-2 agonist enhanced TRPV4 activity and hypersensitivity, while the inhibition of PKA and PKC restrained this effect (99, 107). Also, 5-HT and histamine improved TRPV4-induced hypersensitivity for colorectal distention in mice (108). These results indicate the responses of TRPV4 to inflammatory mediators through GPCR signaling pathway. A selective blockade of TRPV4 was subsequently evident to alleviate intestinal inflammation and pain in TNBS-treated mice (72). Therefore, the pro-hypersensitivity function of TRPV4 during colitis is relatively clear.

### TRPM2

In TNBS-induced rat colitis, the VMR was enhanced and could be ameliorated by an oral administration of TRPM2 antagonist (econazole) (90). However, in TRPM2-knockout (*Trpm2*^−/−^) mice, the TNBS-induced VMR was less severe compared to WT mice (90), showing a potential facilitating role of TRPM2 in VHS.

### TRPM8

Menthol, serving as the agonist for TRPM8, has been applied to relieve abdominal discomfort and pain in traditional Chinese medicine, suggesting that TRPM8 activation can diminish visceral pain perception (12). In TNBS-induced colitis, the colonic mechano-hypersensitivity was remarkably suppressed by a combined adoption of peppermint and caraway oil, which are agonists for TRPM8 (102). Harrington et al. (101) demonstrated that TRPM8 activation restrained the downstream chemosensory and mechanosensory actions of TRPA1 and TRPV1 to agonists in colonic afferents, stating the potential function of TRPM8 for inhibiting TRPV1 and TRPA1. In contrast, it was showed that the TRPM8 agonist (WS-12) enhanced visceral pain response while a pretreatment of TRPM8 antagonist inhibited the hypersensitivity (82). Another study reported that in *Trpm8*^−/−^ mice, VMR only decreased when the pressure level of colorectal distension was quite high; but in *Trpv1*^−/−^ and *Trpv4*^−/−^ mice, VMR was remarkably decreased in all pressure ranges (100). Of note, both bradykinin and histamine were found to suppress TRPM8 mainly via the G-protein subunit Gα which inhibited ion channel activity of TRPM8 (109), indicating the ability of inflammatory mediators to desensitize TRPM8 and inhibit its function. Such a mechanism may account for the TRPM8-associated enhanced-VHS during inflammation.

## TRP Channels in Immune Pathogenesis of IBD

In addition to the roles in inflammatory VHS, the potential engagement of TRP channels in the immune pathogenesis of IBD has been highlighted in human and animal studies (Table 3).

**Table 3:** The function of TRP channels in pathophysiology of colitis models.

**Pro-inflammatory function**			
**TRP channel**	**Colitis model**	**Result**	**References**
TRPV1	DSS-treated mice	Chemically denervation of TRPV1, the antagonist for TRPV1, and the Trpa1-knockout alleviated colitis	(110–114)
	TNBS-treated rat	The antagonist for TRPV1 alleviated colitis	(115)
	TLR-4^−/−^ mice with TNBS-induced colitis	Downregulated TRPV1 expression and alleviated colitis compared to WT mice	(84)
	Toxin-A treated isolated rat ileal segment	Aggravated inflammation which could be enhanced by the agonist or be alleviated by the antagonist for TRPV1	(116)
	T cell-transfer mice colitis	Genetic or pharmacological inhibition of TRPV1 in T cell or colonic tissue resulted in less severe colitis	(117, 118)
TRPA1	DSS-treated mice	The Trpa1-knockout and the antagonist for TRPA1 alleviated colitis	(119)
	TNBS-treated mice	The Trpa1-knockout and the antagonist for TRPA1 alleviated colitis	(119)
TRPV4	DSS-treated mice	The agonist for TRPV4 aggravated colitis and the Trpv4-knockout alleviated colitis	(73, 81)
	TNBS-treated mice	The antagonist for TRPV4 alleviated colitis	(72)
TRPM2	DSS-treated mice	The Trpm2-knockout alleviated colitis	(120)
**Anti-inflammatory function**			
TRPV1	DSS-treated rat	The agonist for TRPV1 alleviated colitis and chemically denervation of TRPV1 aggravated colitis	(121)
	TNBS-treated rat	The agonist for TRPV1 alleviated colitis	(122, 123)
	DNBS-treated mice	The Trpv1-knockout aggravated colitis	(124)
	Oxazolone-treated mice	Chemically denervation of TRPV1 aggravated colitis	(125)
	Iodoacetamide-treated rat	Chemically denervation of TRPV1 aggravated colitis	(126)
	Formalin-treated rabbit	Chemically denervation of TRPV1 aggravated colitis	(127)
TRPA1	DSS-treated mice	The Trpa1-knockout and the antagonist for TRPA1 aggravated colitis; the agonist for TRPA1 alleviated colitis	(71, 85)
	T cell-transfer mice colitis	TRPV1^+^TRPA1^−^ T cells induced more severe colitis compared to TRPV1^+^TRPA1^+^ T cells	(68)
	TNBS-treated mice	The Trpa1-knockout aggravated the fibrosis in colitis	(57, 67)
	DNBS-treated mice	The agonist for TRPA1 alleviated colitis	(85)
TRPM8	TNBS-treated mice	The agonist for TRPM8 alleviated colitis	(77)
	DSS-treated mice	The Trpm8-knockout aggravated colitis and the agonist for TRPM8 alleviated colitis; adoptive transfer of TRPM8^−/−^ macrophages in mice induced more severe colitis compared to WT macrophages	(60, 77)

*DSS, dextran sulfate sodium; TNBS, 2,4,6-trinitrobenzenesulfonic acid; DNBS, dinitrobenzene sulfonic acid; WT, wild-type*.

### TRPV1

The TRPV1^+^ fibers were increased in the colonic mucosa of IBD patients, along with non-neuronal TRPV1 immunoreactivity (65, 66). Further study confirmed an increased expression of TRPV1 in inflamed tissue of active UC patients compared with non-inflamed tissue, being associated with a relapse and continuous activity of disease (64, 67). However, a downregulated expression of TRPV1 was also revealed in colonic biopsies from UC and CD patients (68, 70, 71), and Rizopoulos et al. (70) found no significant correlation between TRPV1 expression and clinical features in UC patients. In experimental colitis models, TRPV1 expression was also found to be altered (68, 78–80, 83, 84, 86) (Table 1). Kihara et al. (110) subcutaneously injected noxious-dose capsaicin into neonatal rats to chemically denervate the TRPV1 channel, revealing that the denervated rats exhibited a lower severity of DSS-induced colitis compared with the control group. Similarly, it was showed that an oral administration of capsazepine (CPZ), which is a specific antagonist for TRPV1, significantly reduced the overall macroscopic epithelial damage in mice colonic tissue after intraperitoneal DSS-administration (111). In *Trpv1*^−/−^ mice, the DSS-induced colitis was less severe (112, 113), and a DSS-associated upregulation of SP-positive fibers was reduced (114), demonstrating a crosstalk between TRPV1 and neurogenic inflammation in colitis. In addition, it was reported that rats with TNBS-induced colitis exhibited a reduction of macroscopic damage score and MPO activity after CPZ enema (115). Recent data pointed that TLR4-knockout mice showed a less inflammatory infiltration and a decreased expression of TRPV1 in TNBS-induced colitis, indicating one possible function of TLR4 for mediating TRPV1 signaling under inflammatory conditions (84). As for other animal models, McVey et al. (116) suggested that an intraluminal administration of capsaicin in isolated ileal segments of rats led to an intestinal inflammation which could be reduced by CPZ. In T-cell-transfer colitis model, the activation of TRPV1 tended to exacerbate the intestinal inflammation, while the colitis was less severe when the TRPV1 in T cell was genetically or pharmacologically inhibited. The pro-inflammatory property of TRPV1 in T cells may be associated with the release of TNF-α, IFN-γ, IL-2, IL-4, IL-10, and IL-17 (117). In another study, capsaicin-induced TRPV1^+^ fibers-denervation ameliorated the intestinal inflammation in the T-cell-transfer colitis model (118), but the suppressive effect of noxious-dose capsaicin pretreatment only existed in 7–8 weeks old mice for several weeks after T-cell transfer, and these mice eventually developed colitis (128). It was further revealed that the severity of TNBS-induced colitis in the TRPV1^+^ fibers-denervated rats was drastically increased within 3–7 days after TNBS administration. Nevertheless, no significant difference of the colitis was found between denervated rats and normal rats in 14–21 days (129), reinforcing the concept that TRPV1^+^ fibers are involved in the early steps of colitis. Taken together, these reports indicate that the activation of TRPV1 in colon is essential for the propagation of intestinal inflammation, and it might be a proximal event in the inflammatory process.

Noteworthy, several publications have reported the protective effects of TRPV1 in experimental colitis. It was exhibited that a local application of capsaicin and exogenous administration of CGRP ameliorated the colonic lesions in TNBS-induced rat colitis (122, 123). Massa et al. (124) stated that *Trpv1*^−/−^ mice exhibited a worse outcome of colitis and lower expression of anti-inflammatory neuropeptides, such as vasoactive intestinal peptide (VIP) and pituitary adenylate cyclase-activating peptide (PACAP), while the NF-κB and STAT3 signaling pathways were demonstrated to be enhanced (130). Moreover, TRPV1 was reported to restrain the initiation and progression of colon cancer (130). In DSS-treated rats, a daily administration of capsaicin was able to reduce the severity of colitis, while a desensitization of TRPV1^+^-fibers dramatically worsened the inflammation (121). A protective role of TRPV1 was also identified in oxazolone-induced mice colitis, iodoacetamide-induced rat acute colitis, and formalin-induced rabbit acute colitis (125–127). The rather ambiguous findings concerning the roles of TRPV1 in colitis required further scrutiny.

Intriguingly, the expression of the TRPV1 was increased in the distal colon and rectum compared to the proximal colon in mice, and similar proximodistal gradient of CGRP/SP was detected (79, 131). Considering the anatomical distribution pattern of UC that often exhibits an ascending inflammation from rectal to proximal colon (4), the increased activity of TRPV1 and neuropeptides in distal colon might give rise to the increased susceptibility of distal colon to colitis and promote the spread of ascending inflammation. Such observations hint at the correlation between the diverse expression of TRP channels in the GI tract and the anatomical distribution pattern of IBD. Differences of microbial composition in certain gut regions and the crosstalk between microbiota and TRP channels are also likely to underlie the IBD anatomical distribution (6, 132). Likewise, it is reasonable to hypothesize that the diverse function of TRP channels in immune cells may be responsible for the distinct pathological pattern of UC and CD. A clear elucidation of this issue can facilitate a better understanding of the TRP channels and pathogenesis involved in IBD.

### TRPA1

The studies regarding the role of TRPA1 in IBD all showed a upregulated TRPA1 expression in the colonic tissue of IBD patients (Table 1). In animal studies, mice with experimental colitis exhibited an increased TRPA1-mediated colonic neuropeptide release, while the experimental colitis appeared to be less severe after the inhibition of TRPA1 by the antagonist or genetic depletion (119). Additional studies suggested a protective role of TRPA1 in the GI tract. Pagano et al. (85) demonstrated that Cannabidivarin, a potent agonist of TRPA1, was able to attenuate the intestinal inflammation in biopsies from pediatric patients with active UC. In dinitrobenzene sulfonic acid (DNBS)- and DSS-treated mice, Cannabidivarin could also ameliorate neutrophil infiltration, intestinal permeability, cytokine production, and alter the dysregulation of gut microbiota (85). Kun et al. (71) reported that the ablation of TRPA1 aggravated DSS-induced colitis and the activation of TRPA1 reduced the release of neuropeptides, cytokines, and chemokines, such as IL-1β and macrophage chemoattractant protein-1 (MCP-1). Further support showed that TRPA1 activation reduced the level of TNF-α in colitis (2). Given that macrophage is the major producer for TNF-α, it may be that the TRPA1 in macrophages can suppress the release of TNF-α and modulate the anti-colitogenic effect, albeit the definite mechanism remains unclear. In addition, some evidence indicated that the expression of TRPA1 was increased in colonic stenotic regions of CD patients. The extent of intestinal inflammation and fibrotic changes in TNBS-treated TRPA1^−/−^ mice were more prominent compare to WT mice and the fibrosis could not be suppressed by inhibitors. The underlying mechanism was considered to be based on the anti-fibrotic role of TRPA1 in intestinal myofibroblasts (74, 75). These observations hint a novel therapeutic target to relieve the fibrosis in IBD.

Whilst considering the highly co-expressive nature of TRPV1 and TRPA1 in colonic afferents, it is interesting to shed light on the interaction between TRPV1 and TRPA1 in colitis. A stimulation of TRPA1 in dorsal root ganglia could result in the activation of PKA and subsequent phosphorylation of TRPV1 (133), while the activation of TRPV1 in afferents could desensitize TRPA1 through phosphatidylinosital biphosphate (PIP_2_) depletion (134). In IBD patients, a vast infiltration of TRPV1^+^TRPA1^+^ T cells had been identified in inflamed colonic tissue (68). Bertin et al. (68) found that TRPV1^+^TRPA1^−^ T cells were able to enhance T-cell receptor-induced Ca^2+^ influx and aggravated intestinal inflammation in IL-10 knockout mice and T-cell-transfer colitis models compared to TRPV1^+^TRPA1^+^ T cells. However, the colitogenic properties of TRPV1^+^TRPA1^−^ T cells were abrogated with pharmacological inhibition or genetic deletion of TRPV1 (68, 117), suggesting that TRPA1 inhibited TRPV1 activity in CD4^+^ T cells and consequently restrained the activity of CD4^+^ T cells. Thus, the role of TRPA1 in colitis could be either protective or damaging.

### TRPV4

The TRPV4 mRNA expression and TRPV4 immunoreactivity in colon were remarkably upregulated in IBD patients (70, 72, 76), in particular, serosal blood vessels with active inflammation were more densely innervated by TRPV4-positive fibers, which often co-localized with the infiltrating CD45^+^ cells (73, 76). Meanwhile, TRPV4 activation could recruit macrophages and other immune cells through the induction of chemokines, such as IL-8 and MCP-1 (73). D'Aldebert et al. (73) indicated the upregulated colonic TRPV4 expression in DSS-treated mice. Intracolonic administration of the TRPV4 agonists (4alpha-phorbol-12,13-didecanoate or GSK1016790A) in mice activated NF-κB and activator protein 1 (AP-1) signaling pathway, resulting in exacerbated DSS-induced colitis and even transiently increased the paracellular permeability of epithelium and blood vessel, while TRPV4-knockout mice conferred a strong resistance to the colitis (73, 81). These results prove the deleterious effects of TRPV4 on mucosal inflammation. Conversely, a systemic or local administration of RN1734, a selective TRPV4 antagonist, remarkably relieved the TNBS-induced colitis (72), suggesting the benefit of attenuating inflammation through blocking TRPV4. The medications aiming at TRPV4 might be capable of alleviating intestinal inflammation in IBD.

### TRPM2

The expression of TRPM2 in distal colon was increased in TNBS-treated rats (90). In *Trpm2*^−/−^ mice, Yamamoto et al. (120) exhibited that the infiltration of immune cells and the severity of intestinal inflammation were ameliorated in DSS-induced colitis. The underlying mechanism might be that the Ca^2+^ influx was impaired in *Trpm2*^−/−^ macrophages, thus affecting the activation of NF-κB pathway (120). This evidence reminds us that TRPM2 can exert pro-inflammatory effects in the colitis via its essential role in macrophages and NF-κB signaling pathway.

### TRPM8

TRPM8 expression was demonstrated to be upregulated in IBD patients and in DSS- or TNBS-treated mice (77). The activation of TRPM8 with icilin significantly attenuated the experimental colitis, but *Trpm8*^−/−^ mice were quite susceptible to colitis (62, 77). It was considered that TRPM8 performed its protective role in the intestine via restraining the release of TNF-α, IL-1, IL-6, and MCP-1, and inducing the release of CGRP (62, 77). TRPM8 activation could also reduce the TRPV1-dependent CGRP release in the gut (77), showing the ability of TRPM8 to suppress the TRPV1-associated inflammatory cascade. The reconstitution of *Trpm8*^−/−^ macrophages in mice exerted a deleterious effect on DSS-induced colitis (60), exhibiting a protective property of TRPM8 in macrophages. These findings reinforce the anti-colitogenic function of TRPM8. Agonists for TRPM8 possibly serve as therapeutic strategies for alleviating intestinal inflammation.

## Possible Factors Behind Prior Contradictory Results

According to aforementioned researches, the roles of a certain type of TRP channels in IBD and experimental colitis tended to be bidirectional or even conflicting.

The human studies mainly concentrated on the expression of TRP channels in the colonic tissue of IBD patients, however, the results of these studies appeared to be contradictory, especially concerning the expression of TRPV1. Actually, TRP channels are widely but anatomically distinctly distributed in various tissues and cell types in the GI tract (6). The expression and function of TRP channels may also be diverse in different subtypes and phases of IBD, and vary among individuals (79). However, the tissue samples of previous studies were acquired at multiple sites of the GI tract and the sample sizes were relatively small. Therefore, further researches that collect sufficient samples from a certain GI region and separately analyze the expression of TRP channels in UC and CD are warranted. Noteworthy, in IBD genome-wide association studies (GWAS), no single nucleotide polymorphism of TRP channel- related genes has been identified in correlation with IBD (135). However, the functions of TRP channels in the GI tract are tightly associated with the content in GI lumen and molecules that possess significant polymorphisms in IBD GWAS, such as TLR4 (136). Additional IBD GWAS studies are needed to uncover specific factors including dietary intake or microbiota in IBD patients in order to explore the definite role of TRP channels polymorphisms in IBD.

Amongst the animal studies regarding TRP channels, researches on TRPV1 and TRPA1 were dominated, thus the majority of conflicting data was related to the functions of these two channels in experimental colitis. Many elements were probably responsible for the paradox.

First, the limitations of the animal models and experimental methods applied in the studies should be considered. Unfortunately, the ideal IBD models that completely mimic the multifactorial chronic disease do not exist and the pathophysiological mechanisms underlying different models are diverse. Also, animals of different strains, species, or ages have their distinct susceptibility to the stimulus, resulting in various demonstrations in experimental colitis (137). Due to the variety of animals and colitis models being used in prior studies, the animal models with distinct characteristics *per se* might accidentally account for the discrepant actions of TRP channels. Meanwhile, the different experimental methodology and drug administration could lead to opposing results. For example, capsaicin, the agonist for TRPV1, has dual effects that the low-dose capsaicin only affects a variable number of TRPV1-expressing nerves, while the high-dose capsaicin results in nerve desensitization (6), indicating the influence of the dose of stimuli on TRP channels. Moreover, the function of TRP channels might be affected by the changes in the microenvironment of the gut induced by agonists or antagonists (138), thus masking the true effects of TRP channels. It was revealed that TRP channels activation could be achieved via overexpression, phosphorylation, or recruitment to the plasma membrane (68). Additional experiments regarding the mode of TRP channels activation induced by specific stimulus may provide a rational view on the interaction between the stimulus and TRP channels.

Second, in addition to the exogenous stimuli applied in studies, there appears to be various endogenous ligands acting on TRP channels, thus influencing the results of experiments. Compounds, such as prostaglandin metabolites, nerve growth factor, and products of oxidative stress can mediate TRPV1 and TRPA1 (5, 95, 139), making it difficult to attribute the results observed in studies to the stimulation of exogenous chemicals or to the stimulation of endogenous mediators. Actually, besides the administration of exogenous stimuli for TRP channels, the activation of TRP channels in IBD is also based on the stimulating effects of multiple endogenous mediators which are synthesized and released within the progress of colitis. Some of these compounds may potentialize TRP channels via the GPCR pathway (5). Hence, it is likely that TRP channels play a role not only in the initiation but also in the regulation of the intestinal inflammation, while the exact mechanism is unclear and needs further explorations.

Third, the functions of neurogenic inflammation and immune responses triggered by TRP channels activation are complicated. The neurogenic inflammation is featured of the release of CGRP and SP, but the effects of these two neuropeptides on intestinal inflammation were not clearly elucidated and tended to be contradictory. The differences in the concentration of neuropeptides and the expression of receptors might contribute to the discrepancy (4). SP was reported to sensitize TRPV1 during colitis and affect the functions of TRPV1 (78), suggesting a possible feedback sensitization loop between neuropeptides and TRP channels. In addition, a range of evidence showed that neuropeptides, such as somatostatin, galanin, opioid peptides, VIP, and PACAP could participate in the inflammation and regulate the inflammatory responses (130, 140). It is warranted to explore whether there is an association between these neuropeptides and TRP channels in colitis. As for immunity, besides the TRP channels-expressing immune cells, some non-immune cells may have TRP channels in the colitis. For example, the expression and function of TRPA1 were identified in fibroblasts which could transform into myofibroblasts and contribute to the regulation of intestinal inflammation (74, 75, 105). However, the definite involvement of myofibroblasts in colitis was poorly understood. Additional explorations are necessary to reveal other TRP channels-expressing cells that play a role in colitis.

Fourth, the TRP channels may interact with various cellular pathways. For instance, the inhibition of TRPV1 could lead to an increased availability of anandamide, and then induced downstream effects on NF-κB and TNF-α and affected bowel motility via the receptor for anandamide (78, 124). Meanwhile, anandamide could also act on TRPV1 and regulate a protection against intestinal inflammation (78, 124), suggesting a potential synergy between TRP channels and other cellular pathways in some settings. Similar to the interaction between TRPV1 and TRPA1, the possible crosstalk between other subtypes of TRP channels is also worth noting. Further studies are warranted to elucidate the comprehensive regulatory network induced by the stimulation of TRP channels in colitis.

In general, the stimulation of TRP channels and a serial cascade of events are like double-edged swords in the intestinal inflammation that exert colitogenic or anti-colitogenic effects in different situations, influenced by a myriad of interactions amongst stimuli, neuropeptides, and the immunity. It is a challenge to figure out the accurate action of a certain type of TRP channels in a specific immune or cellular pathway. Intriguingly, Cohen et al. (22) applied *in vivo* optogenetic strategy to selectively stimulate cutaneous TRPV1^+^-neuron and showed that the afferent activation was based on a nerve reflex. In this study, the activation of TRPV1 was in the absence of other inflammatory stimuli, thus specifically demonstrating the precise role of TRPV1 in the afferents and its immunity-triggering effects. Considering the intense interactions amongst various factors in previous researches, utilizing novel technologies that can efficiently eliminate interferences is a promising strategy for further studies on TRP channels.

## Therapeutic Values of TRP Channels for IBD

As discussed, the effects of TRP channels in IBD have been increasingly appreciated, it is intriguing for researchers to explore their therapeutic values for relieving inflammatory VHS and intestinal inflammation. Pharmacologically, the modulating agents for TRP channels include antagonists and stimulant agonists (141). In particular, antagonists for TRP channels exert a specific effect on modification of ion channel, and stimulant agonists facilitate the desensitization of sensitive afferents (142). However, owing to the wide distribution and various physiological roles of TRP channels within and outside the GI tract (143), the modulation of TRP channels may result in pronounced side effects, such as hyper-thermic effect and impaired injurious-heat perception generated by TRPV1 antagonists (144, 145). Therefore, it is vital to develop the stimulus-specific blockers for TRP channels that specifically act on the aberrant function while sparing the physiological function.

Besides targeting TRP channels directly, it is worth noting that aiming at the stimulus and downstream pathways for GPCRs tends to be another valuable method of restraining the action of TRP channels, especially in the inflammatory process. A novel class of endogenous lipid mediators named resolvin, which are generated from immune cells, such as eosinophils and neutrophils, are of particular interest and have the ability to suppress the function of TRP channels including TRPV1, TRPA1, and TRPV4 (146, 147). The anti-inflammatory effects of resolvin are likely based on the activation of inhibitory GPCR that subsequently suppresses the GPCR-associated sensitization or activation of TRP channels (148), showing the feasibility for inhibiting TRP channels through regulating GPCRs. Overall, treatment strategies targeting TRP channels and their signaling pathways predict a promising future for alleviating the symptoms and improving the prognosis of IBD. More studies are warranted to identify the efficacy and safety of these therapeutic approaches.

**Figure 1:**
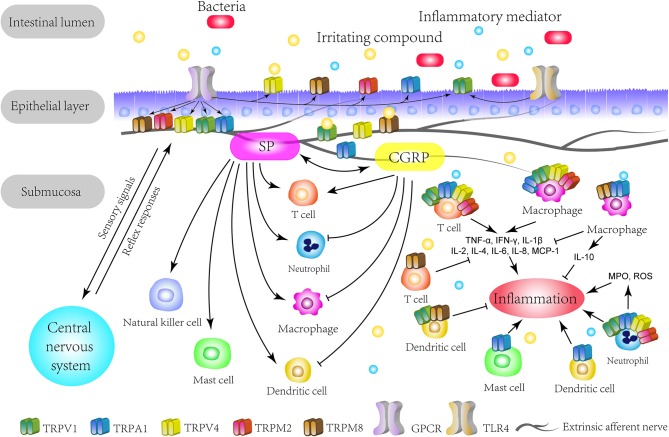
The overview of TRP channels involved in IBD. The TRP channels mainly express themselves on extrinsic primary afferents in the intestine. TRP channels directly detect various stimuli in the intestinal lumen and act as secondary transducers for GPCR. Specially, TRPV1 and TRPA1 can crosstalk with microbiota through TLR4 or in a TLR4-independent manner. Upon activation, TRP channels transduce the sensory signal to the central nervous system and lead to autonomic reflex responses. This mechanism could be enhanced by inflammatory mediators and be responsible for the visceral hypersensitivity on pathological conditions. Meanwhile, the activation of TRP channels triggers neurogenic inflammation with neuropeptides, such as CGRP and SP, which can interact with immune cells. On the other hand, TRP channels express on multiple immune cells and regulate their functions, thus promoting or restraining the initiation or process of inflammation. Therefore, based on the immunomodulatory effects, TRP channels play a role in the immune pathogenesis of IBD. GPCR, G-protein coupled receptors; TLR4, Toll-like receptor 4; SP, substance P; CGRP, calcitonin-gene-related peptide; TNF-α, tumor necrosis factor alpha; IFN-γ, interferon gamma; MCP-1, macrophage chemoattractant protein-1; MPO, myeloperoxidase; ROS, reactive oxygen species.

**Figure 2:**
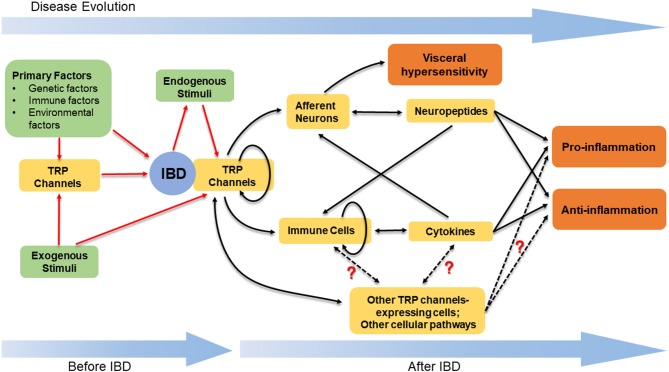
The stimulation of TRP channels and the downstream regulatory network in IBD. TRP channels, together with other primary factors, play a role in the pathogenesis of IBD. The stimulation of TRP channels is based on the exogenous stimuli and the endogenous stimuli. The latter mainly refer to the mediators synthesized and released within the progress of colitis. The activated TRP channels can induce the release of neuropeptides and cytokines, thus leading to the pro- or anti-inflammatory effects. In addition to neurons and immune cells, there are other TRP channels-expressing cells and cellular pathways contributing to regulate the intestinal inflammation, while the definite functions of these cells and pathways are unclear. The complicated crosstalk amongst the neuropeptides, cytokines, TRP channels-expressing cells, and diverse cellular pathways results in the various but elusive effects induced by the stimulation of TRP channels in IBD.

To conclude, TRP channels are not only widely distributed on neurons in the GI tract, functioning as detectors for stimuli and triggers for neurogenic inflammation, but also expressed in multiple immune cells and modulate immune responses (Figure 1). Accumulated evidence has supported an important association between TRP channels and IBD. Although different types of TRP channels exert distinct effects, it is evident that TRP channels are involved in the VHS and the pathogenesis of IBD through a complicated and elusive regulatory network (Figure 2). The inhibition or activation of selected TRP channels can restrain the development of VHS and inflammation in the context of colitis. Therefore, TRP antagonists and agonists tend to constitute an attractive target in IBD treatment and need further attention.

## Author Contributions

YC and HZ reviewed the literature and outlined the overall manuscript. YC wrote the manuscript. JM and MZ helped to write the manuscript. AM and HZ revised the manuscript. HZ supervised the preparation of the draft and edited it and worked as a corresponding author. All authors approved the final version.

### Conflict of Interest

The authors declare that the research was conducted in the absence of any commercial or financial relationships that could be construed as a potential conflict of interest.

## References

[1] Zhang YZ Li YY. Inflammatory bowel disease: pathogenesis. World J Gastroenterol. (2014) 20:91–9. 10.3748/wjg.v20.i1.91 24415861 PMC3886036

[2] Khalil M Alliger K Weidinger C Yerinde C Wirtz S Becker C. Functional role of transient receptor potential channels in immune cells and epithelia. Front Immunol. (2018) 9:174. 10.3389/fimmu.2018.00174 29467763 PMC5808302

[3] Zheng J. Molecular mechanism of TRP channels. Compr Physiol. (2013) 3:221–42. 10.1002/cphy.c120001 23720286 PMC3775668

[4] Engel MA Becker C Reeh PW Neurath MF. Role of sensory neurons in colitis: increasing evidence for a neuroimmune link in the gut. Inflamm Bowel Dis. (2011) 17:1030–3. 10.1002/ibd.21422 20722067

[5] Zielinska M Jarmuz A Wasilewski A Salaga M Fichna J. Role of transient receptor potential channels in intestinal inflammation and visceral pain: novel targets in inflammatory bowel diseases. Inflamm Bowel Dis. (2015) 21:419–27. 10.1097/MIB.0000000000000234 25437822

[6] Holzer P. Transient receptor potential (TRP) channels as drug targets for diseases of the digestive system. Pharmacol Ther. (2011) 131:142–70. 10.1016/j.pharmthera.2011.03.006 21420431 PMC3107431

[7] Inoue R Kurahara LH Hiraishi K. TRP channels in cardiac and intestinal fibrosis. Semin Cell Dev Biol. (2019) 94:40–9. 10.1016/j.semcdb.2018.11.002 30445149

[8] Yang F Zheng J. Understand spiciness: mechanism of TRPV1 channel activation by capsaicin. Protein Cell. (2017) 8:169–77. 10.1007/s13238-016-0353-7 28044278 PMC5326624

[9] Cseko K Beckers B Keszthelyi D Helyes Z. Role of TRPV1 and TRPA1 ion channels in inflammatory bowel diseases: potential therapeutic targets? Pharmaceuticals. (2019) 12:48. 10.3390/ph12020048 30935063 PMC6630403

[10] Vergnolle N. TRPV4: new therapeutic target for inflammatory bowel diseases. Biochem Pharmacol. (2014) 89:157–61. 10.1016/j.bcp.2014.01.005 24440740

[11] Du Q Liao Q Chen C Yang X Xie R Xu J. The role of transient receptor potential vanilloid 1 in common diseases of the digestive tract and the cardiovascular and respiratory system. Front Physiol. (2019) 10:1064. 10.3389/fphys.2019.01064 31496955 PMC6712094

[12] Balemans D Boeckxstaens GE Talavera K Wouters MM. Transient receptor potential ion channel function in sensory transduction and cellular signaling cascades underlying visceral hypersensitivity. Am J Physiol Gastrointest Liver Physiol. (2017) 312:G635–48. 10.1152/ajpgi.00401.2016 28385695

[13] Veldhuis NA Poole DP Grace M McIntyre P Bunnett NW. The G protein-coupled receptor-transient receptor potential channel axis: molecular insights for targeting disorders of sensation and inflammation. Pharmacol Rev. (2015) 67:36–73. 10.1124/pr.114.009555 25361914

[14] Allais L De Smet R Verschuere S Talavera K Cuvelier CA Maes T. Transient receptor potential channels in intestinal inflammation: what is the impact of cigarette smoking? Pathobiology. (2017) 84:1–15. 10.1159/000446568 27388890

[15] Sousa-Valente J Brain SD. A historical perspective on the role of sensory nerves in neurogenic inflammation. Semin Immunopathol. (2018) 40:229–36. 10.1007/s00281-018-0673-1 29616309 PMC5960476

[16] Holzmann B. Antiinflammatory activities of CGRP modulating innate immune responses in health and disease. Curr Protein Pept Sci. (2013) 14:268–74. 10.2174/13892037113149990046 23745695

[17] Assas BM Miyan JA Pennock JL. Cross-talk between neural and immune receptors provides a potential mechanism of homeostatic regulation in the gut mucosa. Mucosal Immunol. (2014) 7:1283–9. 10.1038/mi.2014.80 25183366

[18] Altmayr F Jusek G Holzmann B. The neuropeptide calcitonin gene-related peptide causes repression of tumor necrosis factor-alpha transcription and suppression of ATF-2 promoter recruitment in Toll-like receptor-stimulated dendritic cells. J Biol Chem. (2010) 285:3525–31. 10.1074/jbc.M109.066787 20018859 PMC2823491

[19] Gomes RN Castro-Faria-Neto HC Bozza PT Soares MB Shoemaker CB David JR. Calcitonin gene-related peptide inhibits local acute inflammation and protects mice against lethal endotoxemia. Shock. (2005) 24:590–4. 10.1097/01.shk.0000183395.29014.7c 16317392

[20] Pinho-Ribeiro FA Baddal B Haarsma R O'Seaghdha M Yang NJ Blake KJ. Blocking neuronal signaling to immune cells treats streptococcal invasive infection. Cell. (2018) 173:1083–97. 10.1016/j.cell.2018.04.006 29754819 PMC5959783

[21] Nagashima H Mahlakoiv T Shih HY Davis FP Meylan F Huang Y. Neuropeptide CGRP limits group 2 innate lymphoid cell responses and constrains type 2 inflammation. Immunity. (2019) 51:682–95.e6. 10.1016/j.immuni.2019.06.009 31353223 PMC6801073

[22] Cohen JA Edwards TN Liu AW Hirai T Jones MR Wu J. Cutaneous TRPV1(+) neurons trigger protective innate type 17 anticipatory immunity. Cell. (2019) 178:919–32. 10.1016/j.cell.2019.06.022 31353219 PMC6788801

[23] Talme T Liu Z Sundqvist KG. The neuropeptide calcitonin gene-related peptide (CGRP) stimulates T cell migration into collagen matrices. J Neuroimmunol. (2008) 196:60–6. 10.1016/j.jneuroim.2008.02.007 18423624

[24] Levite M. Neurotransmitters activate T-cells and elicit crucial functions via neurotransmitter receptors. Curr Opin Pharmacol. (2008) 8:460–71. 10.1016/j.coph.2008.05.001 18579442

[25] Li FJ Zou YY Cui Y Yin Y Guo G Lu FG. Calcitonin gene-related peptide is a promising marker in ulcerative colitis. Dig Dis Sci. (2013) 58:686–93. 10.1007/s10620-012-2406-y 23010746

[26] Anand U Yiangou Y Akbar A Quick T MacQuillan A Fox M. Glucagon-like peptide 1 receptor (GLP-1R) expression by nerve fibres in inflammatory bowel disease and functional effects in cultured neurons. PLoS ONE. (2018) 13:e198024. 10.1371/journal.pone.0198024 29813107 PMC5973579

[27] Bossaller C Reither K Hehlert-Friedrich C Auch-Schwelk W Graf K Grafe M. *In vivo* measurement of endothelium-dependent vasodilation with substance P in man. Herz. (1992) 17:284–90. 1282120

[28] Schlereth T Schukraft J Kramer-Best HH Geber C Ackermann T Birklein F. Interaction of calcitonin gene related peptide (CGRP) and substance P (SP) in human skin. Neuropeptides. (2016) 59:57–62. 10.1016/j.npep.2016.06.001 27344069

[29] Suvas S. Role of substance P neuropeptide in inflammation, wound healing, and tissue homeostasis. J Immunol. (2017) 199:1543–52. 10.4049/jimmunol.1601751 28827386 PMC5657331

[30] Green DP Limjunyawong N Gour N Pundir P Dong X. A mast-cell-specific receptor mediates neurogenic inflammation and pain. Neuron. (2019) 101:412–20. 10.1016/j.neuron.2019.01.012 30686732 PMC6462816

[31] Zhao D Kuhnt-Moore S Zeng H Pan A Wu JS Simeonidis S. Substance P-stimulated interleukin-8 expression in human colonic epithelial cells involves Rho family small GTPases. Biochem J. (2002) 368:665–72. 10.1042/bj20020950 12169092 PMC1222994

[32] El-Salhy M Solomon T Hausken T Gilja OH Hatlebakk JG. Gastrointestinal neuroendocrine peptides/amines in inflammatory bowel disease. World J Gastroenterol. (2017) 23:5068–85. 10.3748/wjg.v23.i28.5068 28811704 PMC5537176

[33] Hong HS Hwang DY Park JH Kim S Seo EJ Son Y. Substance-P alleviates dextran sulfate sodium-induced intestinal damage by suppressing inflammation through enrichment of M2 macrophages and regulatory T cells. Cytokine. (2017) 90:21–30. 10.1016/j.cyto.2016.10.002 27750083

[34] Hwang DY Kim S Hong HS. Substance-P ameliorates dextran sodium sulfate-induced intestinal damage by preserving tissue barrier function. Tissue Eng Regen Med. (2018) 15:63–73. 10.1007/s13770-017-0085-7 30603535 PMC6171643

[35] Zhao JF Ching LC Kou YR Lin SJ Wei J Shyue SK. Activation of TRPV1 prevents OxLDL-induced lipid accumulation and TNF-alpha-induced inflammation in macrophages: role of liver X receptor alpha. Mediators Inflamm. (2013) 2013:925171. 10.1155/2013/925171 23878415 PMC3710635

[36] Fernandes ES Liang L Smillie SJ Kaiser F Purcell R Rivett DW. TRPV1 deletion enhances local inflammation and accelerates the onset of systemic inflammatory response syndrome. J Immunol. (2012) 188:5741–51. 10.4049/jimmunol.1102147 22547700

[37] Bertin S de Jong PR Jefferies WA Raz E. Novel immune function for the TRPV1 channel in T lymphocytes. Channels. (2014) 8:479–80. 10.4161/19336950.2014.991640 25530461 PMC4594317

[38] Ghoneum MH Gimzewski JK Ghoneum A Katano H Paw U CN Agrawal A. Inhibition of TRPV1 channel activity in human CD4^+^ T cells by nanodiamond and nanoplatinum liquid, DPV576. Nanomaterials (Basel). (2018) 8:770. 10.3390/nano8100770 30274279 PMC6215208

[39] Jaromi P Garab D Hartmann P Bodnar D Nyiri S Santha P. Capsaicin-induced rapid neutrophil leukocyte activation in the rat urinary bladder microcirculatory bed. Neurourol Urodyn. (2018) 37:690–8. 10.1002/nau.23376 28762564

[40] Acharya N Penukonda S Shcheglova T Hagymasi AT Basu S Srivastava PK. Endocannabinoid system acts as a regulator of immune homeostasis in the gut. Proc Natl Acad Sci USA. (2017) 114:5005–10. 10.1073/pnas.1612177114 28439004 PMC5441729

[41] Yao E Zhang G Huang J Yang X Peng L Huang X. Immunomodulatory effect of oleoylethanolamide in dendritic cells via TRPV1/AMPK activation. J Cell Physiol. (2019) 234:18392–407. 10.1002/jcp.28474 30895621

[42] Baral P Umans BD Li L Wallrapp A Bist M Kirschbaum T Nociceptor sensory neurons suppress neutrophil and γδ T cell responses in bacterial lung infections and lethal pneumonia. Nat Med. (2018) 24:417–26. 10.1038/nm.4501 29505031 PMC6263165

[43] Sahoo SS Majhi RK Tiwari A Acharya T Kumar PS Saha S. Transient receptor potential ankyrin1 channel is endogenously expressed in T cells and is involved in immune functions. Biosci Rep. (2019) 39:BSR20191437. 10.1042/BSR20191437 31488616 PMC6753326

[44] Gouin O L'Herondelle K Lebonvallet N Le Gall-Ianotto C Sakka M Buhe V. TRPV1 and TRPA1 in cutaneous neurogenic and chronic inflammation: pro-inflammatory response induced by their activation and their sensitization. Protein Cell. (2017) 8:644–61. 10.1007/s13238-017-0395-5 28364279 PMC5563280

[45] Zhou Y Han D Follansbee T Wu X Yu S Wang B. Transient receptor potential ankyrin 1 (TRPA1) positively regulates imiquimod-induced, psoriasiform dermal inflammation in mice. J Cell Mol Med. (2019) 23:4819–28. 10.1111/jcmm.14392 31111624 PMC6584593

[46] Romano B Borrelli F Fasolino I Capasso R Piscitelli F Cascio M. The cannabinoid TRPA1 agonist cannabichromene inhibits nitric oxide production in macrophages and ameliorates murine colitis. Br J Pharmacol. (2013) 169:213–29. 10.1111/bph.12120 23373571 PMC3632250

[47] Diogenes A Ferraz CC Akopian AN Henry MA Hargreaves KM. LPS sensitizes TRPV1 via activation of TLR4 in trigeminal sensory neurons. J Dent Res. (2011) 90:759–64. 10.1177/0022034511400225 21393555

[48] De Winter BY Bredenoord AJ Van Nassauw L De Man JG De Schepper HU Timmermans JP. Involvement of afferent neurons in the pathogenesis of endotoxin-induced ileus in mice: role of CGRP and TRPV1 receptors. Eur J Pharmacol. (2009) 615:177–84. 10.1016/j.ejphar.2009.04.055 19445917

[49] Perez-Burgos A Wang L McVey NK Mao YK Ahmadzai M Janssen LJ. The TRPV1 channel in rodents is a major target for antinociceptive effect of the probiotic Lactobacillus reuteri DSM 17938. J Physiol. (2015) 593:3943–57. 10.1113/JP270229 26084409 PMC4575579

[50] Meseguer V Alpizar YA Luis E Tajada S Denlinger B Fajardo O. TRPA1 channels mediate acute neurogenic inflammation and pain produced by bacterial endotoxins. Nat commun. (2014) 5:3125. 10.1038/ncomms4125 24445575 PMC3905718

[51] Li M Fang XZ Zheng YF Xie YB Ma XD Liu XT. Transient receptor potential vanilloid 4 is a critical mediator in LPS mediated inflammation by mediating calcineurin/NFATc3 signaling. Biochem Biophys Res Commun. (2019) 513:1005–12. 10.1016/j.bbrc.2019.04.020 31005256

[52] Scheraga RG Abraham S Niese KA Southern BD Grove LM Hite RD. TRPV4 mechanosensitive ion channel regulates lipopolysaccharide-stimulated macrophage phagocytosis. J Immunol. (2016) 196:428–36. 10.4049/jimmunol.1501688 26597012 PMC4684994

[53] Yin J Michalick L Tang C Tabuchi A Goldenberg N Dan Q. Role of transient receptor potential vanilloid 4 in neutrophil activation and acute lung injury. Am J Respir Cell Mol Biol. (2016) 54:370–83. 10.1165/rcmb.2014-0225OC 26222277

[54] Majhi RK Sahoo SS Yadav M Pratheek BM Chattopadhyay S Goswami C. Functional expression of TRPV channels in T cells and their implications in immune regulation. FEBS J. (2015) 282:2661–81. 10.1111/febs.13306 25903376

[55] Naert R Lopez-Requena A Voets T Talavera K Alpizar YA. Expression and functional role of TRPV4 in bone marrow-derived CD11c(+) cells. Int J Mol Sci. (2019) 20:3378. 10.3390/ijms20143378 31295806 PMC6678969

[56] Wehrhahn J Kraft R Harteneck C Hauschildt S. Transient receptor potential melastatin 2 is required for lipopolysaccharide-induced cytokine production in human monocytes. J Immunol. (2010) 184:2386–93. 10.4049/jimmunol.0902474 20107186

[57] Gershkovitz M Caspi Y Fainsod-Levi T Katz B Michaeli J Khawaled S. TRPM2 mediates neutrophil killing of disseminated tumor cells. Cancer Res. (2018) 78:2680–90. 10.1158/0008-5472.CAN-17-3614 29490946

[58] Mittal M Nepal S Tsukasaki Y Hecquet CM Soni D Rehman J Neutrophil activation of endothelial cell-expressed TRPM2 mediates transendothelial neutrophil migration and vascular injury. Circ Res. (2017) 121:1081–91. 10.1161/CIRCRESAHA.117.311747 28790198 PMC5640489

[59] Melzer N Hicking G Gobel K Wiendl H. TRPM2 cation channels modulate T cell effector functions and contribute to autoimmune CNS inflammation. PLoS ONE. (2012) 7:e47617. 10.1371/journal.pone.0047617 23077651 PMC3470594

[60] Khalil M Babes A Lakra R Forsch S Reeh PW Wirtz S. Transient receptor potential melastatin 8 ion channel in macrophages modulates colitis through a balance-shift in TNF-alpha and interleukin-10 production. Mucosal immunol. (2016) 9:1500–13. 10.1038/mi.2016.16 26982596

[61] Juergens UR Engelen T Racke K Stober M Gillissen A Vetter H. Inhibitory activity of 1,8-cineol (eucalyptol) on cytokine production in cultured human lymphocytes and monocytes. Pulm Pharmacol Ther. (2004) 17:281–7. 10.1016/j.pupt.2004.06.002 15477123

[62] de Jong PR Takahashi N Peiris M Bertin S Lee J Gareau MG. TRPM8 on mucosal sensory nerves regulates colitogenic responses by innate immune cells via CGRP. Mucosal Immunol. (2015) 8:491–504. 10.1038/mi.2014.82 25269705 PMC4382463

[63] Kume H Tsukimoto M. TRPM8 channel inhibitor AMTB suppresses murine T-cell activation induced by T-cell receptor stimulation, concanavalin A, or external antigen re-stimulation. Biochem Biophys Res Commun. (2019) 509:918–24. 10.1016/j.bbrc.2019.01.004 30642628

[64] Akbar A Yiangou Y Facer P Brydon WG Walters JR Anand P Expression of the TRPV1 receptor differs in quiescent inflammatory bowel disease with or without abdominal pain. Gut. (2010) 59:767–74. 10.1136/gut.2009.194449 20551462

[65] Luo C Wang Z Mu J Zhu M Zhen Y Zhang H. Upregulation of the transient receptor potential vanilloid 1 in colonic epithelium of patients with active inflammatory bowel disease. Int J Clin Exp Pathol. (2017) 10:11335–44. 31966488 PMC6965867

[66] Yiangou Y Facer P Dyer NH Chan CL Knowles C Williams NS. Vanilloid receptor 1 immunoreactivity in inflamed human bowel. Lancet. (2001) 357:1338–39. 10.1016/S0140-6736(00)04503-7 11343743

[67] Toledo-Maurino JJ Furuzawa-Carballeda J Villeda-Ramirez MA Fonseca-Camarillo G Meza-Guillen D Barreto-Zuniga R. The transient receptor potential vanilloid 1 is associated with active inflammation in ulcerative colitis. Mediators Inflamm. (2018) 2018:6570371. 10.1155/2018/6570371 30150894 PMC6087567

[68] Bertin S Aoki-Nonaka Y Lee J de Jong PR Kim P Han T. The TRPA1 ion channel is expressed in CD4+ T cells and restrains T-cell-mediated colitis through inhibition of TRPV1. Gut. (2017) 66:1584–96. 10.1136/gutjnl-2015-310710 27325418 PMC5173457

[69] Keszthelyi D Troost FJ Jonkers DM Helyes Z Hamer HM Ludidi S. Alterations in mucosal neuropeptides in patients with irritable bowel syndrome and ulcerative colitis in remission: a role in pain symptom generation? Eur J Pain. (2013) 17:1299–306. 10.1002/j.1532-2149.2013.00309.x 23529955

[70] Rizopoulos T Papadaki-Petrou H Assimakopoulou M. Expression profiling of the transient receptor potential vanilloid (TRPV) channels 1, 2, 3 and 4 in mucosal epithelium of human ulcerative colitis. Cells. (2018) 7:61. 10.3390/cells7060061 29914124 PMC6025154

[71] Kun J Szitter I Kemeny A Perkecz A Kereskai L Pohoczky K. Upregulation of the transient receptor potential ankyrin 1 ion channel in the inflamed human and mouse colon and its protective roles. PLoS ONE. (2014) 9:e108164. 10.1371/journal.pone.0108164 25265225 PMC4180273

[72] Fichna J Mokrowiecka A Cygankiewicz AI Zakrzewski PK Malecka-Panas E Janecka A. Transient receptor potential vanilloid 4 blockade protects against experimental colitis in mice: a new strategy for inflammatory bowel diseases treatment? Neurogastroenterol Motil. (2012) 24:e557–60. 10.1111/j.1365-2982.2012.01999.x 22882778

[73] D'Aldebert E Cenac N Rousset P Martin L Rolland C Chapman K. Transient receptor potential vanilloid 4 activated inflammatory signals by intestinal epithelial cells and colitis in mice. Gastroenterology. (2011) 140:275–85. 10.1053/j.gastro.2010.09.045 20888819

[74] Hiraishi K Kurahara LH Sumiyoshi M Hu YP Koga K Onitsuka M. Daikenchuto (Da-Jian-Zhong-Tang) ameliorates intestinal fibrosis by activating myofibroblast transient receptor potential ankyrin 1 channel. World J Gastroenterol. (2018) 24:4036–53. 10.3748/wjg.v24.i35.4036 30254408 PMC6148431

[75] Kurahara LH Hiraishi K Hu Y Koga K Onitsuka M Doi M. Activation of myofibroblast TRPA1 by steroids and pirfenidone ameliorates fibrosis in experimental Crohn's disease. Cell Mol Gastroenterol Hepatol. (2018) 5:299–318. 10.1016/j.jcmgh.2017.12.005 29552620 PMC5852292

[76] Brierley SM Page AJ Hughes PA Adam B Liebregts T Cooper NJ. Selective role for TRPV4 ion channels in visceral sensory pathways. Gastroenterology. (2008) 134:2059–69. 10.1053/j.gastro.2008.01.074 18343379 PMC2504007

[77] Ramachandran R Hyun E Zhao L Lapointe TK Chapman K Hirota CL. TRPM8 activation attenuates inflammatory responses in mouse models of colitis. Proc Natl Acad Sci USA. (2013) 110:7476–81. 10.1073/pnas.1217431110 23596210 PMC3645521

[78] Lapointe TK Basso L Iftinca MC Flynn R Chapman K Dietrich G. TRPV1 sensitization mediates postinflammatory visceral pain following acute colitis. Am J Physiol Gastrointest Liver Physiol. (2015) 309:G87–99. 10.1152/ajpgi.00421.2014 26021808

[79] Engel MA Khalil M Mueller-Tribbensee SM Becker C Neuhuber WL Neurath MF. The proximodistal aggravation of colitis depends on substance P released from TRPV1-expressing sensory neurons. J Gastroenterol. (2012) 47:256–65. 10.1007/s00535-011-0495-6 22080974

[80] Matsumoto K Lo MW Hosoya T Tashima K Takayama H Murayama T. Experimental colitis alters expression of 5-HT receptors and transient receptor potential vanilloid 1 leading to visceral hypersensitivity in mice. Lab Invest. (2012) 92:769–82. 10.1038/labinvest.2012.14 22330338

[81] Matsumoto K Yamaba R Inoue K Utsumi D Tsukahara T Amagase K. Transient receptor potential vanilloid 4 channel regulates vascular endothelial permeability during colonic inflammation in dextran sulphate sodium-induced murine colitis. Br J Pharmacol. (2018) 175:84–99. 10.1111/bph.14072 29053877 PMC5740260

[82] Hosoya T Matsumoto K Tashima K Nakamura H Fujino H Murayama T. TRPM8 has a key role in experimental colitis-induced visceral hyperalgesia in mice. Neurogastroenterol Motil. (2014) 26:1112–21. 10.1111/nmo.12368 24832648

[83] Yang M Wang J Yang C Han H Rong W Zhang G. Oral administration of curcumin attenuates visceral hyperalgesia through inhibiting phosphorylation of TRPV1 in rat model of ulcerative colitis. Mol Pain. (2017) 13:2071437104. 10.1177/1744806917726416 28812431 PMC5562337

[84] Wu Y Wang Y Wang J Fan Q Zhu J Yang L. TLR4 mediates upregulation and sensitization of TRPV1 in primary afferent neurons in 2,4,6-trinitrobenzene sulfate-induced colitis. Mol pain. (2019) 15:2069333502. 10.1177/1744806919830018 30672380 PMC6378437

[85] Pagano E Romano B Iannotti FA Parisi OA D'Armiento M Pignatiello S. The non-euphoric phytocannabinoid cannabidivarin counteracts intestinal inflammation in mice and cytokine expression in biopsies from UC pediatric patients. Pharmacol Res. (2019) 149:104464. 10.1016/j.phrs.2019.104464 31553934

[86] Miranda A Nordstrom E Mannem A Smith C Banerjee B Sengupta JN. The role of transient receptor potential vanilloid 1 in mechanical and chemical visceral hyperalgesia following experimental colitis. Neuroscience. (2007) 148:1021–32. 10.1016/j.neuroscience.2007.05.034 17719181 PMC2128774

[87] Salameh E Meleine M Gourcerol G Do RJ Do RJ Legrand R. Chronic colitis-induced visceral pain is associated with increased anxiety during quiescent phase. Am J Physiol Gastrointest Liver Physiol. (2019) 316:G692–700. 10.1152/ajpgi.00248.2018 30735453

[88] Yang J Li Y Zuo X Zhen Y Yu Y Gao L. Transient receptor potential ankyrin-1 participates in visceral hyperalgesia following experimental colitis. Neurosci Lett. (2008) 440:237–41. 10.1016/j.neulet.2008.05.093 18583045

[89] Li Q Guo CH Chowdhury MA Dai TL Han W. TRPA1 in the spinal dorsal horn is involved in post-inflammatory visceral hypersensitivity: *in vivo* study using TNBS-treated rat model. J Pain Res. (2016) 9:1153–60. 10.2147/JPR.S118581 27980434 PMC5144908

[90] Matsumoto K Takagi K Kato A Ishibashi T Mori Y Tashima K. Role of transient receptor potential melastatin 2 (TRPM2) channels in visceral nociception and hypersensitivity. Exp Neurol. (2016) 285:41–50. 10.1016/j.expneurol.2016.09.001 27616276

[91] Kimball ES Prouty SP Pavlick KP Wallace NH Schneider CR Hornby PJ. Stimulation of neuronal receptors, neuropeptides and cytokines during experimental oil of mustard colitis. Neurogastroenterol Motil. (2007) 19:390–400. 10.1111/j.1365-2982.2007.00939.x 17509021

[92] Makimura Y Ito K Kuwahara M Tsubone H. Augmented activity of the pelvic nerve afferent mediated by TRP channels in dextran sulfate sodium (DSS)-induced colitis of rats. J Vet Med Sci. (2012) 74:1007–13. 10.1292/jvms.11-0547 22498929

[93] Phillis BD Martin CM Kang D Larsson H Lindstrom EA Martinez V. Role of TRPV1 in high-threshold rat colonic splanchnic afferents is revealed by inflammation. Neurosci Lett. (2009) 459:57–61. 10.1016/j.neulet.2009.04.051 19406204

[94] Mitrovic M Shahbazian A Bock E Pabst MA Holzer P. Chemo-nociceptive signalling from the colon is enhanced by mild colitis and blocked by inhibition of transient receptor potential ankyrin 1 channels. Br J Pharmacol. (2010) 160:1430–42. 10.1111/j.1476-5381.2010.00794.x 20590633 PMC2938814

[95] Kogure Y Wang S Tanaka K Hao Y Yamamoto S Nishiyama N. Elevated H2 O2 levels in trinitrobenzene sulfate-induced colitis rats contributes to visceral hyperalgesia through interaction with the transient receptor potential ankyrin 1 cation channel. J Gastroenterol Hepatol. (2016) 31:1147–53. 10.1111/jgh.13226 26574143

[96] Vermeulen W De Man JG De Schepper HU Bult H Moreels TG Pelckmans PA. Role of TRPV1 and TRPA1 in visceral hypersensitivity to colorectal distension during experimental colitis in rats. Eur J Pharmacol. (2013) 698:404–12. 10.1016/j.ejphar.2012.10.014 23099257

[97] Brierley SM Hughes PA Page AJ Kwan KY Martin CM O'Donnell TA. The ion channel TRPA1 is required for normal mechanosensation and is modulated by algesic stimuli. Gastroenterology. (2009) 137:2084–95. 10.1053/j.gastro.2009.07.048 19632231 PMC2789877

[98] Cattaruzza F Spreadbury I Miranda-Morales M Grady EF Vanner S Bunnett NW. Transient receptor potential ankyrin-1 has a major role in mediating visceral pain in mice. Am J Physiol Gastrointest Liver Physiol. (2010) 298:G81–91. 10.1152/ajpgi.00221.2009 19875705 PMC2806099

[99] Grant AD Cottrell GS Amadesi S Trevisani M Nicoletti P Materazzi S. Protease-activated receptor 2 sensitizes the transient receptor potential vanilloid 4 ion channel to cause mechanical hyperalgesia in mice. J Physiol. (2007) 578:715–33. 10.1113/jphysiol.2006.121111 17124270 PMC2151332

[100] Mueller-Tribbensee SM Karna M Khalil M Neurath MF Reeh PW Engel MA. Differential contribution of TRPA1, TRPV4 and TRPM8 to colonic nociception in mice. PLoS ONE. (2015) 10:e128242. 10.1371/journal.pone.0128242 26207981 PMC4514604

[101] Harrington AM Hughes PA Martin CM Yang J Castro J Isaacs NJ. A novel role for TRPM8 in visceral afferent function. Pain. (2011) 152:1459–68. 10.1016/j.pain.2011.01.027 21489690

[102] Adam B Liebregts T Best J Bechmann L Lackner C Neumann J. A combination of peppermint oil and caraway oil attenuates the post-inflammatory visceral hyperalgesia in a rat model. Scand J Gastroenterol. (2006) 41:155–60. 10.1080/00365520500206442 16484120

[103] Vecchi BL Marcuzzi A Tricarico PM Zanin V Girardelli M Bianco AM Curcumin and inflammatory bowel disease: potential and limits of innovative treatments. Molecules. (2014) 19:21127–53. 10.3390/molecules191221127 25521115 PMC6271352

[104] Jones RR Xu L Gebhart GF. The mechanosensitivity of mouse colon afferent fibers and their sensitization by inflammatory mediators require transient receptor potential vanilloid 1 and acid-sensing ion channel 3. J Neurosci. (2005) 25:10981–9. 10.1523/JNEUROSCI.0703-05.2005 16306411 PMC6725875

[105] Yang Y Wang S Kobayashi K Hao Y Kanda H Kondo T. TRPA1-expressing lamina propria mesenchymal cells regulate colonic motility. JCI Insight. (2019) 4:e122402. 10.1172/jci.insight.122402 31045572 PMC6538323

[106] Feng CC Yan XJ Chen X Wang EM Liu Q Zhang LY. Vagal anandamide signaling via cannabinoid receptor 1 contributes to luminal 5-HT modulation of visceral nociception in rats. Pain. (2014) 155:1591–604. 10.1016/j.pain.2014.05.005 24813296

[107] Cenac N Altier C Chapman K Liedtke W Zamponi G Vergnolle N. Transient receptor potential vanilloid-4 has a major role in visceral hypersensitivity symptoms. Gastroenterology. (2008) 135:937–46, 941–6. 10.1053/j.gastro.2008.05.024 18565335

[108] Cenac N Altier C Motta JP D'Aldebert E Galeano S Zamponi GW. Potentiation of TRPV4 signalling by histamine and serotonin: an important mechanism for visceral hypersensitivity. Gut. (2010) 59:481–8. 10.1136/gut.2009.192567 20332520

[109] Zhang X Mak S Li L Parra A Denlinger B Belmonte C. Direct inhibition of the cold-activated TRPM8 ion channel by Galphaq. Nat Cell Biol. (2012) 14:851–8. 10.1038/ncb2529 22750945 PMC3428855

[110] Kihara N de la Fuente SG Fujino K Takahashi T Pappas TN Mantyh CR. Vanilloid receptor-1 containing primary sensory neurones mediate dextran sulphate sodium induced colitis in rats. Gut. (2003) 52:713–9. 10.1136/gut.52.5.713 12692058 PMC1773638

[111] Kimball ES Wallace NH Schneider CR D'Andrea MR Hornby PJ. Vanilloid receptor 1 antagonists attenuate disease severity in dextran sulphate sodium-induced colitis in mice. Neurogastroenterol Motil. (2004) 16:811–8. 10.1111/j.1365-2982.2004.00549.x 15601431

[112] Fujino K Fuente SGDL Pappas TN Mantyh CR Dextran sulfate sodium-induced enterocolitis is attenuated in vanilloid receptor-1 knockout mice. Gastroenterology. (2003) 124:A141–2. 10.1016/S0016-5085(03)80700-X

[113] Szitter I Pozsgai G Sandor K Elekes K Kemeny A Perkecz A. The role of transient receptor potential vanilloid 1 (TRPV1) receptors in dextran sulfate-induced colitis in mice. J Mol Neurosci. (2010) 42:80–8. 10.1007/s12031-010-9366-5 20411352

[114] Utsumi D Matsumoto K Tsukahara T Amagase K Tominaga M Kato S. Transient receptor potential vanilloid 1 and transient receptor potential ankyrin 1 contribute to the progression of colonic inflammation in dextran sulfate sodium-induced colitis in mice: Links to calcitonin gene-related peptide and substance P. J Pharmacol Sci. (2018) 136:121–32. 10.1016/j.jphs.2017.12.012 29478714

[115] Fujino K Takami Y de la Fuente SG Ludwig KA Mantyh CR. Inhibition of the vanilloid receptor subtype-1 attenuates TNBS-colitis. J Gastrointest Surg. (2004) 8:842–7, 847–8. 10.1016/j.gassur.2004.07.011 15531237

[116] McVey DC Vigna SR. The capsaicin VR1 receptor mediates substance P release in toxin A-induced enteritis in rats. Peptides. (2001) 22:1439–46. 10.1016/S0196-9781(01)00463-6 11514026

[117] Bertin S Aoki-Nonaka Y de Jong PR Nohara LL Xu H Stanwood SR. The ion channel TRPV1 regulates the activation and proinflammatory properties of CD4(+) T cells. Nat Immunol. (2014) 15:1055–63. 10.1038/ni.3009 25282159 PMC4843825

[118] Gad M Pedersen AE Kristensen NN Fernandez CF Claesson MH. Blockage of the neurokinin 1 receptor and capsaicin-induced ablation of the enteric afferent nerves protect SCID mice against T-cell-induced chronic colitis. Inflamm Bowel Dis. (2009) 15:1174–82. 10.1002/ibd.20902 19326358

[119] Engel MA Leffler A Niedermirtl F Babes A Zimmermann K Filipovic MR. TRPA1 and substance P mediate colitis in mice. Gastroenterology. (2011) 141:1346–58. 10.1053/j.gastro.2011.07.002 21763243

[120] Yamamoto S Shimizu S Kiyonaka S Takahashi N Wajima T Hara Y. TRPM2-mediated Ca2+influx induces chemokine production in monocytes that aggravates inflammatory neutrophil infiltration. Nat Med. (2008) 14:738–47. 10.1038/nm1758 18542050 PMC2789807

[121] Okayama M Tsubouchi R Kato S Takeuchi K. Protective effect of lafutidine, a novel histamine H2-receptor antagonist, on dextran sulfate sodium-induced colonic inflammation through capsaicin-sensitive afferent neurons in rats. Dig Dis Sci. (2004) 49:1696–704. 10.1023/B:DDAS.0000043389.96490.76 15573930

[122] Evangelista S Tramontana M. Involvement of calcitonin gene-related peptide in rat experimental colitis. J Physiol Paris. (1993) 87:277–80. 10.1016/0928-4257(93)90017-N 8136795

[123] Goso C Evangelista S Tramontana M Manzini S Blumberg PM Szallasi A. Topical capsaicin administration protects against trinitrobenzene sulfonic acid-induced colitis in the rat. Eur J Pharmacol. (1993) 249:185–90. 10.1016/0014-2999(93)90431-G 8287899

[124] Massa F Sibaev A Marsicano G Blaudzun H Storr M Lutz B. Vanilloid receptor (TRPV1)-deficient mice show increased susceptibility to dinitrobenzene sulfonic acid induced colitis. J Mol Med. (2006) 84:142–6. 10.1007/s00109-005-0016-2 16389550

[125] Barada KA Kafrouni MI Khoury CI Saade NE Mourad FH Szabo SS. Experimental colitis decreases rat jejunal amino acid absorption: role of capsaicin sensitive primary afferents. Life sci. (2001) 69:3121–31. 10.1016/S0024-3205(01)01418-7 11758837

[126] Lee J Yamamoto T Kuramoto H Kadowaki M. TRPV1 expressing extrinsic primary sensory neurons play a protective role in mouse oxazolone-induced colitis. Auton Neurosci. (2012) 166:72–6. 10.1016/j.autneu.2011.07.008 21855422

[127] Reinshagen M Patel A Sottili M Nast C Davis W Mueller K. Protective function of extrinsic sensory neurons in acute rabbit experimental colitis. Gastroenterology. (1994) 106:1208–14. 10.1016/0016-5085(94)90011-6 7513664

[128] Claesson MH. Limited effect of capsaicin in T-cell transfer colitis. Inflamm Bowel Dis. (2011) 17:E121. 10.1002/ibd.21789 21674729

[129] Reinshagen M Patel A Sottili M French S Sternini C Eysselein VE. Action of sensory neurons in an experimental at colitis model of injury and repair. Am J Physiol. (1996) 270:G79–86. 10.1152/ajpgi.1996.270.1.G79 8772504

[130] Vinuesa AG Sancho R Garcia-Limones C Behrens A Ten DP Calzado MA. Vanilloid receptor-1 regulates neurogenic inflammation in colon and protects mice from colon cancer. Cancer Res. (2012) 72:1705–16. 10.1158/0008-5472.CAN-11-3693 22396497

[131] Matsumoto K Kurosawa E Terui H Hosoya T Tashima K Murayama T. Localization of TRPV1 and contractile effect of capsaicin in mouse large intestine: high abundance and sensitivity in rectum and distal colon. Am J Physiol Gastrointest Liver Physiol. (2009) 297:G348–60. 10.1152/ajpgi.90578.2008 19497956

[132] Bilotta AJ Cong Y. Gut microbiota metabolite regulation of host defenses at mucosal surfaces: implication in precision medicine. Precis Clin Med. (2019) 2:110–9. 10.1093/pcmedi/pbz008 31281735 PMC6598739

[133] Spahn V Stein C Zollner C. Modulation of transient receptor vanilloid 1 activity by transient receptor potential ankyrin 1. Mol Pharmacol. (2014) 85:335–44. 10.1124/mol.113.088997 24275229

[134] Akopian AN Ruparel NB Jeske NA Hargreaves KM. Transient receptor potential TRPA1 channel desensitization in sensory neurons is agonist dependent and regulated by TRPV1-directed internalization. J Physiol. (2007) 583:175–93. 10.1113/jphysiol.2007.133231 17584831 PMC2277224

[135] Jostins L Ripke S Weersma RK Duerr RH McGovern DP Hui KY. Host-microbe interactions have shaped the genetic architecture of inflammatory bowel disease. Nature. (2012) 491:119–24. 10.1038/nature11582 23128233 PMC3491803

[136] Ao R Wang Y Zhnag DR Du YQ. Role of TLR4 rs4986790A>G and rs4986791C>T polymorphisms in the risk of inflammatory bowel disease. Gastroenterol Res Pract. (2015) 2015:141070. 10.1155/2015/141070 26089865 PMC4451775

[137] Krieglstein CF Cerwinka WH Laroux FS Salter JW Russell JM Schuermann G. Regulation of murine intestinal inflammation by reactive metabolites of oxygen and nitrogen: divergent roles of superoxide and nitric oxide. J Exp Med. (2001) 194:1207–18. 10.1084/jem.194.9.1207 11696587 PMC2195977

[138] Belmaati MS Diemer S Hvarness T Baumann K Pedersen AE Christensen RE. Antiproliferative effects of TRPV1 ligands on nonspecific and enteroantigen-specific T cells from wild-type and Trpv1 KO mice. Inflamm Bowel Dis. (2014) 20:1004–14. 10.1097/MIB.0000000000000039 24788222

[139] Akbar A Yiangou Y Facer P Walters JR Anand P Ghosh S. Increased capsaicin receptor TRPV1-expressing sensory fibres in irritable bowel syndrome and their correlation with abdominal pain. Gut. (2008) 57:923–9. 10.1136/gut.2007.138982 18252749 PMC2564830

[140] Banvolgyi A Palinkas L Berki T Clark N Grant AD Helyes Z. Evidence for a novel protective role of the vanilloid TRPV1 receptor in a cutaneous contact allergic dermatitis model. J Neuroimmunol. (2005) 169:86–96. 10.1016/j.jneuroim.2005.08.012 16188326

[141] Baraldi PG Preti D Materazzi S Geppetti P. Transient receptor potential ankyrin 1 (TRPA1) channel as emerging target for novel analgesics and anti-inflammatory agents. J Med Chem. (2010) 53:5085–107. 10.1021/jm100062h 20356305

[142] Szallasi A Cortright DN Blum CA Eid SR. The vanilloid receptor TRPV1: 10 years from channel cloning to antagonist proof-of-concept. Nat Rev Drug Discov. (2007) 6:357–72. 10.1038/nrd2280 17464295

[143] Wu LJ Sweet TB Clapham DE. International union of basic and clinical pharmacology. LXXVI. Current progress in the mammalian TRP ion channel family. Pharmacol Rev. (2010) 62:381–404. 10.1124/pr.110.002725 20716668 PMC2964900

[144] Wong GY Gavva NR. Therapeutic potential of vanilloid receptor TRPV1 agonists and antagonists as analgesics: Recent advances and setbacks. Brain Res Rev. (2009) 60:267–77. 10.1016/j.brainresrev.2008.12.006 19150372

[145] Voight EA Kort ME. Transient receptor potential vanilloid-1 antagonists: a survey of recent patent literature. Expert Opin Ther Pat. (2010) 20:1107–22. 10.1517/13543776.2010.497756 20586701

[146] Xu ZZ Zhang L Liu T Park JY Berta T Yang R. Resolvins RvE1 and RvD1 attenuate inflammatory pain via central and peripheral actions. Nat Med. (2010) 16:592–7, 1–597. 10.1038/nm.2123 20383154 PMC2866054

[147] Serhan CN. Pro-resolving lipid mediators are leads for resolution physiology. Nature. (2014) 510:92–101. 10.1038/nature13479 24899309 PMC4263681

[148] Williams JT Ingram SL Henderson G Chavkin C von Zastrow M Schulz S. Regulation of μ-opioid receptors: desensitization, phosphorylation, internalization, and tolerance. Pharmacol Rev. (2013) 65:223–54. 10.1124/pr.112.005942 23321159 PMC3565916

